# Assessing the dose of regadenoson required to transiently alter blood-brain barrier integrity in patients with infiltrating gliomas

**DOI:** 10.1093/noajnl/vdaf041

**Published:** 2025-02-15

**Authors:** Stuart A Grossman, Carlos G Romo, Xiaobu Ye, Brian Kral, Roy E Strowd, Glenn Lesser, Catalina Raymond, Michaella Iacoboni, Serena Desideri, Joy Fisher, Neeraja Danda, Benjamin M Ellingson

**Affiliations:** Department of Oncology, The Johns Hopkins University School of Medicine, Baltimore, Maryland, USA; Department of Neurology, The Johns Hopkins University School of Medicine, Baltimore, Maryland, USA; Department of Neurosurgery, The Johns Hopkins University School of Medicine, Baltimore, Maryland, USA; Department of Radiology, The Johns Hopkins University School of Medicine, Baltimore, Maryland, USA; Department of Internal Medicine, Hematology and Oncology, Wake Forest University School of Medicine, Wake Forest, North Carolina, USA; Department of Internal Medicine, Hematology and Oncology, Wake Forest University School of Medicine, Wake Forest, North Carolina, USA; UCLA Brain Tumor Imaging Laboratory, Department of Radiological Sciences, Center for Computer Vision and Imaging Biomarkers, Los Angeles, California, USA; Department of Oncology, The Johns Hopkins University School of Medicine, Baltimore, Maryland, USA; Department of Oncology, The Johns Hopkins University School of Medicine, Baltimore, Maryland, USA; Department of Oncology, The Johns Hopkins University School of Medicine, Baltimore, Maryland, USA; Department of Oncology, The Johns Hopkins University School of Medicine, Baltimore, Maryland, USA; UCLA Brain Tumor Imaging Laboratory, Department of Radiological Sciences, Center for Computer Vision and Imaging Biomarkers, Los Angeles, California, USA

**Keywords:** adenosine agonist, blood-brain barrier, glioma, gadolinium, magnetic resonance imaging (MRI)

## Abstract

**Background:**

The blood-brain barrier (BBB) severely limits the delivery of therapeutic agents to the brain. Regadenoson, a Food and Drug Administration-approved adenosine A2 agonist, transiently increases BBB permeability in rodents to a 70 kDa dextran. This multi-institutional, NIH-funded study examined regadenoson’s ability to transiently alter BBB permeability in patients with gliomas.

**Methods:**

Adults with supratentorial gliomas at low risk for regadenoson complications were treated with 1 of the 7 dose levels known to be safe in humans. Successful BBB disruption was defined as a 10-fold increase in vascular permeability (*K*^*trans*^) relative to historic benchmarks. This was assessed by dynamic contrast-enhanced perfusion on magnetic resonance imaging in normal-appearing white matter (NAWM) changes in NAWM and non-enhancing tumors were also quantified using contrast-enhanced T1 subtraction maps.

**Results:**

Seven patients <45 years old with low-grade gliomas were accrued before the study was prematurely closed. Regadenoson was well tolerated. Following regadenoson, *K*^*trans*^ in NAWM did not reach the targeted *K*^*trans*^ threshold (0.04 min^−1^). Normalized subtraction maps of contrast-enhanced T1-weighted MR signal intensity in NAWM did increase an average of 74% ± 22% (*P* = .016) after regadenoson.

**Conclusions:**

No dose of regadenoson significantly elevated *K*^*trans*^ in NAWM. However, the subtraction maps suggest that regadenoson may lead to a measurable change in gadolinium flux. This coupled with strong preclinical data suggests further investigation is warranted. Noninvasive quantification of BBB permeability in patients is feasible and could evaluate different regadenoson schedules, combination therapies, and other approaches to modify BBB permeability which is critical to improving outcomes in patients with brain tumors.

**Clinical Trial Registration:**

NCI Protocol #: Adult brain tumor consortium 1804, ClinicalTrials.gov Identifier: *NCT03971734,* Agent(s): Regadenoson, NSC # 811401, commercially available.

**Key Points**

- Regadenoson transiently disrupts BBB integrity in rodents.
- Escalating doses were administered to humans without major changes in BBB integrity.
- This study design is useful to noninvasively assess novel approaches to modify BBB integrity in humans.

**Importance of Study**

Improving the outcome of patients with primary and metastatic brain tumors is highly dependent upon improving the delivery of systemically administered therapies into non-enhancing regions of the brain. Regadenoson, an FDA-approved adenosine agonist, transiently disrupts the blood-brain barrier (BBB) in rodents allowing a 70 kDa dextran to penetrate normal brain parenchyma. Here, we report the first study conducted in humans to examine escalating doses of regadenoson using the *K*^*trans*^ of gadolinium in the non-enhancing brain as the primary endpoint. Although no dramatic changes were seen in BBB integrity after regadenoson, this noninvasive study design provides an important example of how one can evaluate alternate regadenoson schedules or other novel approaches to modify BBB permeability which is crucial to improving the outcome of patients with primary and metastatic brain tumors.

Despite decades of efforts to improve survival in patients with glioblastoma, the median survival of patients with this cancer is about 15 months, the 5-year survival rate is less than 5%, and the cure rate approaches zero.^1,2^ There are many reasons why improving survival has been much more difficult in brain cancers than in other malignancies. These tumors diffusely infiltrate large regions of the brain rendering this tumor surgically incurable. Radiation doses and fields are limited by the sensitivity of the normal brain to this treatment modality. In addition, these cancers have been relatively unresponsive to most chemotherapy drugs, targeted agents, and immunotherapies that have improved outcomes in many systemic solid tumors.

A critical factor that distinguishes the brain from other cancer sites is the presence of the blood-brain barrier (BBB). The presence of a BBB in invertebrate animals with a distributed nervous system and its evolution from a neuronal to an endothelial barrier over millions of years highlights its importance in neural function.^3^ The BBB is critical for maintaining the homeostasis of the brain microenvironment and protecting the brain from infections and toxins. However, it does such a remarkable job that over 98% of pharmaceutical agents currently approved by the Food and Drug Authority are unable to penetrate an intact BBB.^4,5^ It is very efficient in highly restricting the delivery of therapeutic doses of systemically administered chemotherapeutic drugs in patients with primary and metastatic brain tumors.^4^ As a result, patients with systemic cancers who are responding to effective oral or intravenous chemotherapy are at high risk for isolated recurrences within the central nervous system where drug levels are too low to be effective.^6,7^ The efficiency of the BBB also provides important insight into why therapies targeting known pathways and mutations in brain tumors have very limited effects in this patient population.

The BBB is a potentially modifiable barrier. Its integrity can be compromised by exposure to temperature extremes, trauma, surgery, infectious agents, or radiation therapy and can be somewhat restored using glucocorticoids or VEGF-targeted agents. Clinical and laboratory researchers have tried for years to enhance the delivery of therapeutic agents into brain tumors. Three general approaches have been used. The first involves bypassing the BBB by administering agents directly into brain tumors using needles or intrathecal or convection-enhanced delivery catheters, or by placing biodegradable chemotherapy-laden polymers into the resection cavity at the time of surgery.^8,9^ The second relies on increasing concentrations of drugs in the blood supplying brain tumors by selectively catheterizing intracranial vessels for local intra-arterial infusions or administering very high doses of systemic chemotherapy (eg, methotrexate in primary CNS lymphoma).^9,10^ The third approach rests on transiently disrupting the BBB using techniques such as intra-arterial hypertonic mannitol or focused ultrasound followed by the administration of systemic chemotherapy.^8,11^ Unfortunately, each of these approaches has significant limitations, most target limited brain volumes that do not encompass the full tumor volume, and to date, these have not significantly improved survival.^8,12^

An ideal approach to improving outcomes with glioblastoma or preventing brain metastases would safely, reproducibly, and transiently increase the permeability of the entire BBB. Such an agent could be co-administered with systemically administered therapeutic agents allowing them to reach higher concentrations within the entire brain parenchyma. Vasoactive peptides have been shown to transiently alter the integrity of the BBB in preclinical settings. Co-administration of bradykinin analogs in animal models yielded increased carboplatin concentrations in brain tumor tissue without serious toxicities.^13,14^ However, bradykinin analogs combined with carboplatin in patients with recurrent brain tumors did not result in therapeutic benefits in these patients.^15^

Adenosine is another vasoactive peptide that appears to play an important role in regulating the integrity of the BBB.^16–19^ Preclinical studies have documented its ability to transiently open the BBB to a 70 kDa dextran, regulate multidrug-resistant transporters (such as P-glycoprotein pumps), and increase the paracellular transmigration of lymphocytes into the central nervous system.^19,20^ Adenosine-related changes are controlled by 2 of its 4 G- protein-coupled receptors (A1 and A2A) which either inhibit or stimulate downstream activation. The A2A receptor exhibits high expression and functionality within the heart and brain and is an important regulator of local vasodilation. Regadenoson is an FDA-approved adenosine A2A receptor agonist that is widely used in patients with suspected cardiac disease who are unable to participate in a standard exercise stress test.^21^

Preclinical studies in mice and rats have demonstrated that adenosine A2A receptor agonists dramatically increase BBB permeability to a 70 kDa dextran molecule.^16^ Additional studies in non-tumor bearing rats have shown that co-administration of regadenoson and temozolomide results in a 60% increase in temozolomide brain concentrations without altering the systemic pharmacology of temozolomide.^22^ These findings prompted clinical studies of regadenoson using brain SPECT and CT scans to evaluate alterations in CNS permeability to standard imaging agents. Unfortunately, these studies did not reveal a detectable change in the permeability of the BBB to the systemically administered radioactive ligands or iodinated contrast agents.^23,24^ In a small surgical study of patients with recurrent high-grade gliomas who underwent clinically indicated debulking surgery, microdialysis catheters were placed in the peritumoral region. Patients then received a standard dose of oral temozolomide and the following day another dose of temozolomide followed by regadenoson.^24^ Intraparenchymal brain levels of temozolomide were measured after oral temozolomide alone and after temozolomide with regadenoson. No significant differences were found in temozolomide concentrations in the brain. It is important to note that the dose of regadenoson used in these clinical studies was approved by the FDA for cardiac stress tests. The optimal dose of regadenoson to alter the integrity of the BBB in humans is unknown. Interestingly, the dose required to transiently modify the permeability of the BBB is quite different in mice and rats, and in these species, there appears to be a “sweet spot” for the administered dose where doses higher or lower than this were less effective.^16^ We hypothesize that the BBB of both rodents and humans are likely to be sensitive to adenosine agonists but that the FDA-approved dose of regadenoson for cardiac stress testing is either too high or too low to transiently open the BBB in humans. Fortunately, the clinical studies of regadenoson that led to FDA approval for its cardiac indication have nicely documented the maximum tolerated doses of this drug in humans in both the standing and recumbent positions.

This preliminary data led to the pilot study reported in this manuscript. This study was funded by the National Cancer Institute and conducted by the multi-institutional Adult Brain Tumor Consortium (ABTC). The primary objective of this study was to identify a dose of regadenoson that would transiently alter the integrity of the BBB in patients with gliomas. To do this, we explored doses of regadenoson that are known to be well below and well above the FDA-approved dose for cardiac stress testing. Each of these doses had previously been found to be safe in clinical studies leading to FDA approval for cardiac indications.^25–27^ Vascular permeability was estimated after administration of regadenoson using imaging measurements of the forward flux rate constant, *K*^*trans*^, of gadolinium. This was obtained using dynamic contrast-enhanced (DCE) perfusion magnetic resonance imaging (MRI). DCE-MRI is a well-established technique that has been used to evaluate BBB permeability for over 20 years.^28–38^ This technique has served as a primary endpoint in imaging-specific trials.^33,34^ Additionally, we assessed the change in gadolinium contrast accumulation by quantifying the change in T1-weighted signal intensity on contrast-enhanced T1-weighted digital subtraction maps or “T1 subtraction maps.”^34–38^ These imaging measurements were used to assess gadolinium accumulation in normal-appearing brain tissue within the contralateral hemisphere as well as alterations in permeability in the brain adjacent to tumor (BAT) and within the contrast-enhancing tumor regions.

## Methods

This study was reviewed and approved by the National Cancer Institute’s Brain Malignancy Steering Committee and the Institutional Review Board of each participating ABTC member institution. This study was available to each institution participating in the National Cancer Institute’s funded ABTC. The 7 patients participating were accrued at the Johns Hopkins and Wake Forest Universities. The primary objective of this study was to identify a dose of regadenoson, in the range shown to be safe, that increased gadolinium *K*^*trans*^ by more than 10 times the values reported in the literature within normal-appearing brain parenchyma (NAWM) in patients with gliomas. There were 2 secondary objectives in this study. The first was to identify a dose of regadenoson that substantially alters the normalized, contrast-enhanced MRI signal intensity (using T1 subtraction maps) in (a) T2 or FLAIR bright but non-enhancing BAT and (b) in the contrast-enhancing tumor. The second was to evaluate toxicities associated with regadenoson administered to patients with gliomas.

**Figure 1.**
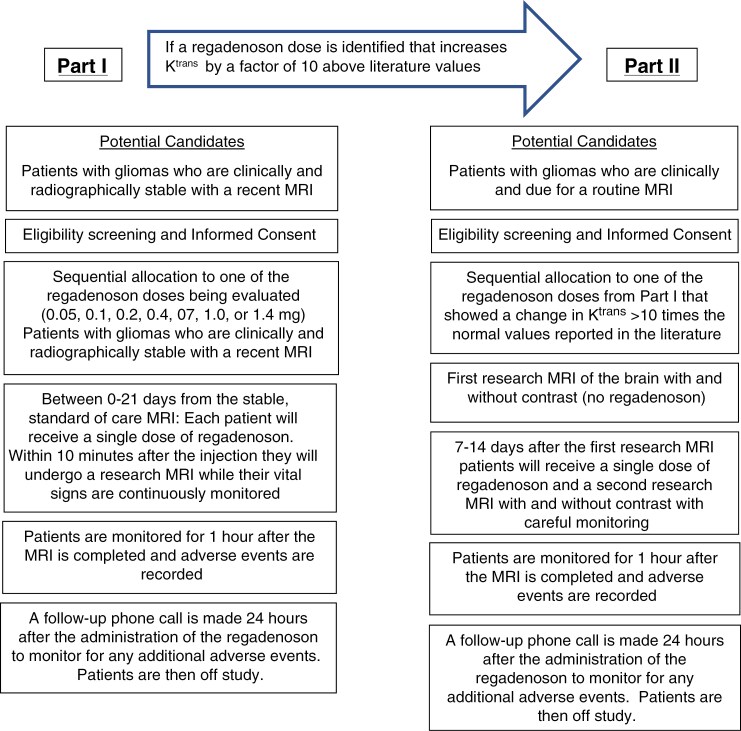
Study schema for Part I and Part II

This study was designed to be conducted in 2 separate parts. Part 1 aimed to screen 7 doses of regadenoson known to be safe in humans for any sign that this agent is facilitating the entry of gadolinium into normal brain parenchyma. To do this, a recently completed routine follow-up MRI scan was used as a baseline and was compared to a research MRI scan that was performed after the patient received regadenoson and gadolinium. If one or more doses of regadenoson were found to significantly increase *K*^*trans*^, Part II of the study would be opened. Part II would employ more rigorous methods where 2 research MRIs would be done in each patient—the first with gadolinium alone and the second after gadolinium and regadenoson. Both Part I and Part II are described below. However, as none of the regadenoson doses tested in Part I met the targeted changes in *K*^*trans*^, Part II was never initiated. A more complete description of the 2 parts of this study is presented below and in Figure 1.

### Part I

Potential candidates for this study were identified with a stable routine follow-up MRI brain with and without gadolinium. They underwent eligibility screening and signed informed consent. They were then sequentially allocated to one of the dose levels being evaluated (see Table 1), Between 0 and 21 days from their stable standard of care MRI they received a single dose of regadenoson and within 10 minutes underwent a research MRI while their vital signs were continuously monitored and a cardiologist was present. One hour after the MRI was completed and all adverse events were recorded, patients were allowed to go home, a follow-up phone call was made 2 hours later, and the patient was officially off study.

**Table 1.** Patient Characteristics and Regadenoson Doses

Patient number	Dose mg	Age/sex race	Histology/grade IDH status	Last surgery	Last radiation	Last ChemoRx	KPS	Enhancing tumor
1	0.05	23 M C	Oligo II IDH-m	2018 GTR	None	None	100	None
2	0.10	45 M C	Oligo/Astro III IDH-?	2012 GTR	2012	2013	100	None
3	0.20	37 F H	Astro II IDH-m	2019 STR	2019	2020	100	None
4	0.40	40 M C	Astro II IDH-m	2018 GTR	None	None	100	None
5	0.70	33 M C	Astro III IDH-m	2017 GTR	2017	2018	80	None
6	1.00	36 F C	Oligo II IDH-m	2019 GTR	None	None	100	None
7	1.40	42 F C	Oligo III IDH-m	2006 GTR	2006	2007	90	None

Dose, Regadenoson dose administered, KPS, Karnofsky Performance Status, Oligo, oligodendroglioma, Astro, astrocytoma, IDH-m, IDH mutated, IDH-?, IDH unknown, GTR, gross total resection, C, Caucasian, H, Hispanic, ChemoRx, chemotherapy.

NOA-D-24-00317R1.

### Part II

Potential candidates were patients who were very likely to have stable MRI scans. Rather than obtaining a standard follow-up MRI, patients enrolled in this part of the study were to have their first research scan with and without contrast but without regadenoson. The details of this scan are described below in the section titled Imaging Methods. They would then be sequentially allocated to one of the dose levels of regadenoson from Part I of the study which showed a change in *K*^*trans*^ of more than 10 times the normal value reported in the literature. Seven to fourteen days later they would have their second research MRI with and without gadolinium after a single dose of regadenoson. Within 10 minutes of regadenoson administration, they would have their MRI while vital signs were continuously monitored and a cardiologist was present. They were to be monitored for 1 hour after the MRI was completed and all adverse events would be recorded. A follow-up phone call would be made 2 hours later to monitor for additional adverse events and at that time the patient would be officially off study.

### Patient Selection

Patients were carefully selected for this non-therapeutic study to minimize risks from the administration of regadenoson. Eligible patients were young, without known cardiac disease, and not taking potentially neurotoxic medications as part of their routine medical care in case Regadenoson opened the BBB and allowed these medications access to the central nervous system is provided in Supplementary Material. Furthermore, every effort was made to ensure that selected patients did not have rapidly progressive tumors as this would complicate the analysis of the efficacy of regadenoson which relied on changes between a baseline scan and the later scan with regadenoson.

Patients who were eligible for this study were required to have a prior histologic diagnosis of a glioma of any grade, be 18–45 years of age, have a KPS of ≥80, be able to provide informed consent, and undergo an MRI with contrast. There were no restrictions regarding the number of prior therapies or relapses but they were required to have serial routine follow-up MRIs that did not reveal progressive disease for over 2 months before entering this study. In addition, they had to be without progressive symptoms or signs of tumor progression. A detailed description of the eligibility and exclusion criteria and a list of prohibited potentially neurotoxic medications are provided in Supplementary Table 1.

### Regadenoson Administration

Seven doses of regadenoson were to be studied in Part I. These included the FDA-approved dose for cardiac studies (0.4 mg) 3 doses below (0.05, 0.1, and 0.2 mg), and 3 doses above the FDA-approved dose (0.7, 1.0, and 1.4 mg). Each of these doses had been shown to be safe in the pre-FDA approval studies of regadenoson for cardiac stress testing.^25–27^ These doses were administered to patients in the supine position and with a cardiologist present. Doses higher than 1.4 mg have been associated with hypotension and other adverse events. The study planned to enroll a maximum of 5 patients at each of these 7 dose levels. If a minimum of 3 out of 5 patients achieved a 10-fold increase in *K*^*trans*^ for gadolinium in normal brain at any dose levels of regadenoson, Part II of the study was to be initiated. Part II of the study was designed to provide a higher level of evidence that the observed drug-induced alterations in BBB integrity were real.

Patients were instructed to avoid consumption of any products containing methylxanthines, including caffeinated coffee, tea, or other caffeinated beverages, caffeine-containing drug products, aminophylline, and theophylline for at least 12 hours before a scheduled scan. Patients were asked not to take anything by mouth for 4–6 hours before testing. Prior to regadenoson administration, each patient was evaluated by the study cardiologist for symptoms or signs consistent with cardiopulmonary exclusion criteria. A cardiovascular and pulmonary exam, vital signs (heart rate, blood pressure, respiratory rate, oxygen saturation, temperature), and a 12-lead electrocardiogram were obtained prior to treatment. A single dose of regadenoson at the assigned dose level was then administered under the supervision of the cardiologist. Institutional research pharmacies provided a single syringe containing the assigned regadenoson dose for each study participant. Regadenoson was administered as a rapid (~10 seconds) injection into a peripheral vein using a 22 gauge or larger catheter or needle, followed immediately by a 5 mL saline flush. For regadenoson doses of 0.7 or 1.0 mg, regadenoson was administered by a slower injection (~20 seconds). For the highest regadenoson dose of 1.4 mg, administration took approximately 3 times longer than the standard dose (~30 seconds). The time of the injection was accurately recorded. The 5 mL flush of normal saline solution was to be administered at the same rate as the dose of regadenoson.

The research MRI scan was performed following the administration of regadenoson along with continuous telemetry monitoring of vital signs and 12-lead electrocardiography. This MRI was performed on a similar machine as the pre-enrollment eligibility MRI scan, using the same administered dose of gadolinium and acquisition parameters. Vital signs (HR, BP, and SpO2) were recorded before the start of the MRI, and every 20 minutes after starting. The research MRI included DCE perfusion MRI for estimation of *K*^*trans*^ using standard doses of gadolinium and regadenoson at the assigned study dose.

Vital signs (blood pressure, heart rate, respiratory rate) and a 12-lead ECG were taken prior to and after regadenoson administration and after MRI. All MRI scanners used were capable of evaluating vital signs with continuous telemetry to be monitored for potential adverse reactions by the study cardiologist. A full range of emergency equipment was available. Antihistamines were also available as were other medications in the event of an allergy. Adenosine receptor agonists, including regadenoson, may cause dyspnea, bronchoconstriction, and respiratory compromise. Appropriate bronchodilator therapy and resuscitative measures were available. Additionally, aminophylline is a competitive adenosine receptor antagonist used to terminate the persistent pharmacodynamic effects of regadenoson. Aminophylline was available if required clinically and was to be administered in doses ranging from 50 to 250 mg by slow intravenous injection.

### Imaging Methods

The imaging study was designed to be conducted in 2 parts. Part I of the study was a screening evaluation to determine if there was initial evidence of activity at any of the 7 regadenoson doses being studied. This was determined by the primary evaluation criteria/endpoint which was defined as *K*^*trans*^* *> 0.04 min^−1^, or a *K*^*trans*^ more than 10x normal-appearing brain (contralateral to the tumor) where the BBB integrity pre-regadenoson is assumed to be normal (*K*^*trans*^ ~ 0.004 min^−1^)^30,32,34,36^ This was chosen with the presumption that opening a relatively normal BBB is required for this approach to significantly change the outcomes in patients with primary and metastatic brain tumors. The secondary evaluation endpoint was designed to evaluate change in normalized, contrast-enhanced MRI signal intensity without and with the administration of regadenoson. Additional evaluations included: (i) post-regadenoson *K*^*trans*^ and (ii) change in contrast-enhanced MRI signal intensity in: (a) BAT (i.e. T2 hyperintensity without observed contrast enhancement) and (b) the region of contrast-enhancing tumor if present. Although the imaging was performed at the ABTC institutions accruing patients, all of the post-processing of DCE-MRI and post-processing of contrast-enhanced T1-weighted digital subtraction was performed centrally by the ABTC Imaging Core (PI: Ben Ellingson).

Part II of this proposed research was designed to utilize advanced imaging to confirm that regadenoson has a significant effect on the BBB using a more comprehensive imaging approach at doses that are initially determined to have activity from Part I. Five additional patients were to be studied at each regadenoson dose that met the desired criteria/endpoint of a *K*^*trans*^* *> 0.04 min^−1^ within contralateral normal-appearing brain following regadenoson administration in Part I of the study. In these cohorts, the full research imaging protocol was to be utilized in both the pre- and post-regadenoson studies to allow a direct comparison of all imaging parameters in both the pre-regadenoson and post-regadenoson settings to be directly compared. Part II of this study was not opened given that Part I did not identify a regadenoson dose that had sufficient impact on BBB permeability.

### DCE Perfusion MRI

DCE-MRI included 2 distinct measurements: 1) pre-contrast T1 mapping and 2) dynamic T1-weighted image acquisition during contrast injection. Pre-contrast T1 mapping was to be performed using a series of spoiled gradient echo T1-weighted images (eg, FLASH or SPGR) collected with 5 different flip angles (2, 10, 15, 20, and 30 deg). No normalization filters were turned on (eg, SCIC or PURE) and acceleration factors (eg, ASSET or iPAT) were limited to 2x or less. The Ernst angle formula was then used to estimate the voxel-wise T1 of the tissue. Following T1 mapping, a dynamic spoiled gradient echo T1-weighted sequence with a flip angle of 20 °C and equivalent echo and repetition times was employed before, during, and for 8–10 minutes after injection of a gadolinium-based contrast agent. The following contrast agents and their molecular weights were approved to be used for this study: Magnevist (gadopentetate dimeglumine, MW 1164 g/mol), OmniScan (gadodiamide, MW 592 g/mol), Dotarem (gadoterate meglumine, MW 949 g/mol), ProHance (gadoteridol, MW 559 g/mol), or Gadavist (gadobutrol, MW 605 g/mol). The Extended Tofts model was used to estimate *K*^*trans*^ for all image voxels, which shows similar performance to other pharmacokinetic models in areas of normal blood flow and volume.^28^*K*^*trans*^ estimated in a spherical region of interest approximately 2 cm in diameter in an area of normal-appearing white matter in the contralateral hemisphere to the area of the tumor was used as the primary measure of BBB permeability. The standardized MRI protocol is illustrated in Supplementary Table 2.

### Contrast-Enhanced T1-Weighted Digital Subtraction

The secondary measurement of change in gadolinium contrast accumulation was measured by quantifying change in T1-weighted signal intensity on contrast-enhanced T1-weighted digital subtraction maps or “T1 subtraction maps.”^33,39,40^ Briefly, parameter-matched, pre- and post-contrast T1-weighted images were first intensity normalized (Z-scores) and bias-field corrected, then registered using a 6-degree-of-freedom, rigid body transformation. Following registration of pre- and post-contrast images, post-contrast T1-weighted images were subtracted voxel-by-voxel from pre-contrast T1-weighted images, generating the final T1 subtraction maps. Gadolinium accumulation in normal-appearing brain tissue within the contralateral hemisphere on the MRI scan prior to regadenoson (standard MRI documenting no change in the tumor activity) was compared with a research MRI performed after regadenoson administration. Anatomic images and T1 subtraction maps obtained before and after regadenoson were subsequently registered to each other and subtracted, resulting in parametric maps quantifying the change in contrast-enhanced, T1-weighted signal intensity occurring as a result of treatment.

### Statistical Considerations

This trial was designed as an open-label, multicenter study to identify a regadenoson dose that could increase gadolinium *K*^*trans*^ by more than 10 times the values reported in the literature within the normal-appearing brain parenchyma (part I) and could substantially alter the normalized, contrast-enhanced MRI signal intensity in normal-appearing tissues (part II). There were 7 pre-specified dose levels of interest. Safety was not a main consideration of the study since the 7 doses are within the safe range of regadenoson as determined in prior studies.^25,27^ Five participants were to be accrued at each dose level to achieve above 90% statistical power to discriminate a clinically desired goal of 80% of patients having *K*^*trans*^ > 0.04 min^−1^ within normal-appearing tissue following regadenoson administration against a 20% for the dose selection (Part I). Patient enrollment was open to 7 dose cohorts simultaneously. For Part II of this study to be opened a minimum of one effective dose had to be identified in part I. Five additional patients were to be enrolled in each dose being selected for part II of the study. Per trial design, the accrual of 35 to 70 participants was expected to be needed to complete both Parts I and II of this study.

### Limitations of This Study

This clinical research study opened in 2020 and closed in 2022. Accrual was slow as this non-therapeutic study did not offer any potential benefit to patients, the eligibility criteria were restrictive, the imaging procedures needed to be standardized among participating institutions, patients received the equivalent of a cardiac stress test as they entered the MRI scanner, and a cardiologist was required to be present to administer the regadenoson and monitor the patient. There were 2 major unanticipated challenges that this study faced. First, this study opened as the COVID-19 pandemic blossomed and when the conduct of non-therapeutic studies at many institutions was slowed or even halted. Additionally, the National Cancer Institute decided in 2021 that federal funds for brain tumor research should prioritize preclinical research. This resulted in the administrative closure of the ABTC and the premature closure of its clinical trials. Other potential limitations to the interpretation of this study include the diversity of available contrast agents and MRI machines at different ABTC institutions and the choice of the selected *K*^*trans*^ endpoint for this study.

## Results

Despite the challenges outlined above, one patient was accrued to each of the 7 planned regadenoson dose levels. Due to the limited number of patients enrolled, the patient characteristics data are presented descriptively and the planned statistical analysis was revised to account for the small patient numbers (Table 1). The median age of these patients was 37 years (range 23 to 44 years). Six patients were Caucasian and one was Hispanic. Four subjects were male and 3 were female. Their median Karnofsky performance status score was 100 (range 80–100). Six of the seven patients had IDH-mutant oligodendrogliomas or astrocytomas and the IDH status in one participant was unknown. Three patients had grade 3 tumors documented at the time of their last surgery. None of the patients had measurable enhancing disease when they were accrued in this study. Five of the seven patients were on anti-seizure medications. Their last surgery was listed as a gross total resection in 6 patients and as a subtotal resection in the seventh patient. Their last surgical dates ranged from 2006 to 2019 (median 2018). Radiation had been administered to 4 of the 7 patients (ending from 2006 to 2019) and 4 had also received chemotherapy (ie, temozolomide or procarbazine) that ended from 2007 to 2020. All patients were undergoing surveillance MR imaging and examinations without active anti-cancer therapy for their tumor when they joined this study. These highly selected young patients all tolerated regadenoson treatment without adverse events.

### Imaging Results

**Figure 2.**
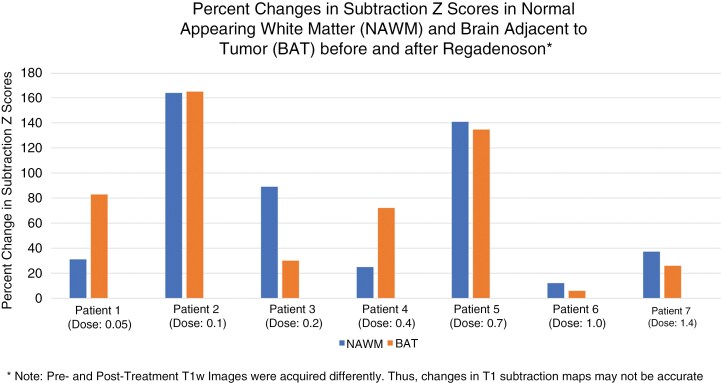
Percent changes in subtraction Z scores in normal-appearing white matter (NAWM) and brain adjacent to tumor (BAT) before and after regadenoson. Pre- and post-treatment T1w images were acquired differently. Thus, changes in T1 subtraction maps may not be accurate.

**Figure 3.**
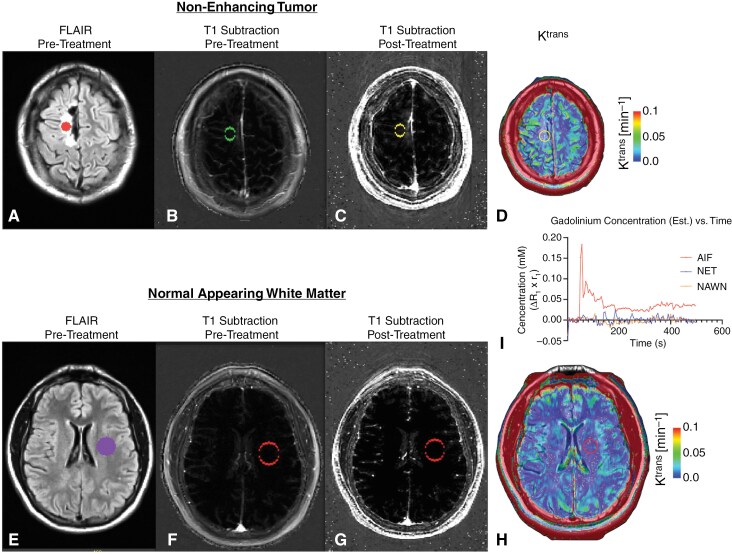
Example of T1 subtraction and DCE results after administration of regadenoson. (A) pretreatment FLAIR images showing region of interest (ROI) selection in areas of T2 hyperintensity consistent with non-enhancing tumor (NET). (B) pretreatment T1-weighted digital subtraction maps, created by subtracting post-contrast T1-weighted images from pre-contrast T1-weighted images, in areas of non-enhancing tumor. (C) Post-treatment T1-weighted digital subtraction maps in areas of non-enhancing tumor. (D) Post-treatment dynamic contrast-enhanced (DCE) perfusion magnetic resonance imaging (MRI) estimates of K^trans^, the forward flux or rate of contrast extravasation, and a surrogate of vascular permeability, in areas of non-enhancing tumor. (E) pretreatment FLAIR images showing ROI selection in areas of normal-appearing white matter (NAWM). (F) pretreatment T1-weighted digital subtraction maps in areas of NAWM. (G) Post-treatment T1-weighted digital subtraction maps in areas of NAWM. (H) Post-treatment DCE perfusion MRI estimates of K^trans^ in areas of NAWM. Estimated contrast agent concentration versus time for the arterial input function (AIF), ROI within the NET, and ROI within NAWM. Areas of NET and NAWM show almost no change in concentration after treatment.

Following the administration of regadenoson, the *K*^*trans*^ measurements in NAWM averaged 1.13 × 10^−3^ ± 0.44 × 10^-3^ (SEM) min^−1^, which is considerably lower than the target *K*^*trans*^ threshold of 0.04 min^−1^ to show a meaningful effect on BBB permeability (Table 2). Consistent with this observation, visual inspection of the post-regadenoson, post-contrast T1-weighted images did not reveal visible changes in contrast enhancement within the contralateral normal brain or the BAT region. However, normalized subtraction maps of contrast-enhanced T1-weighted MR signal intensity in NAWM increased by an average of 74.0% ± 22.4% (SEM; *P* = .0163) when evaluated across all subjects, suggesting that there was a small but measurable change in gadolinium flux after administration of regadenoson (Figures 2 and 3). No dose-dependent effects were observed. Despite these findings, the study failed to meet the pre-specified target *K*^*trans*^ in NAWM and non-enhancing tumors.

**Table 2.** Changes in K-Trans and Z-Scores in Non-Enhancing and Normal Appearing White Matter After Regadenoson Administration

	Non-enhancing tumor					Normal appearing white matter				
Subject # and Regadenoson dose	Post-treatment K^trans^	Pretreatment T1 subtraction [Z-score]	Post-treatment T1 subtraction [Z-score]	Difference in contrast enhancement (D-C)	Difference in contrast enhancement %	Post-treatment K^trans^	Pretreatment T1 subtraction [Z-Score]	Post-treatment T1 subtraction [Z-Score]	Difference in contrast enhancement (D-C)	Difference in contrast enhancement %
#1–0.05 mg	2.39 × 10^−3^	−0.38	−0.5	−0.12	30.7	1.28 × 10^−3^	−0.43	−0.78	−0.36	83.5
#2–0.10 mg	1.34 × 10^−4^	−0.15	−0.42	−0.25	164.7	5.24 × 10^−4^	−0.16	−0.43	-0.27	165.2
#3–0.20 mg	4.76 × 10^−4^	−0.39	−0.49	−0.1	25	3.62 × 10^−3^	−0.42	−0.55	−0.13	30.8
#4–0.40 mg	2.22 × 10^−4^	−0.26	−0.48	−0.23	89.4	5.77 × 10^−4^	−0.31	−0.53	−0.22	72.2
#5–0.70 mg	1.74 × 10^−4^	−0.22	−0.54	−0.32	141.6	2.01 × 10^−4^	−0.25	−0.59	−0.34	135
#6–1.00 mg	1.31 × 10^−4^	−0.5	−0.56	−0.06	12.2	1.09 × 10^−3^	−0.46	−0.49	−0.03	5.5
#7–1.40 mg	2.56 × 10^−4^	−0.31	−0.42	−0.12	37.2	6.21 × 10^−4^	−0.36	−0.46	−0.09	25.6

^**^Note that Pre- and Post-Treatment T1w Images were acquired differently, so change in T1 subtraction maps might not be accurate (except for patient #6).

## Discussion

Conventional surgery and radiation are important treatment modalities for patients with high-grade gliomas. However, there are limits to the amount of brain tissue that can be safely removed or irradiated and these cancers are relatively resistant to radiation. As a result, recurrences routinely occur both inside and outside of the radiation field and the cure rate following these local treatment modalities remains close to zero. During the past several decades there have been extensive efforts to improve patient outcomes by adding chemotherapy, targeted agents, antiangiogenic drugs, and immunotherapy approaches that prolong survival in other solid tumors. The results have been disappointing. Only one chemotherapeutic agent, temozolomide, has been identified to prolong survival in patients with glioblastoma. Adding temozolomide to radiation extends median survival by about 4 months (from 12 to 16 months). The primary benefit of temozolomide is seen in patients with MGMT methylated glioblastomas where the median survival is increased from 15 to 24 months. However, even in these patients, death from recurrent glioblastoma is universal. In 60% of patients with truly MGMT unmethylated glioblastomas, the benefit from the addition of temozolomide to radiation is very limited. Next Generation Sequencing (NGS) studies have shown that high-grade gliomas harbor many of the same mutations and molecular pathway alterations seen in other systemic malignancies.^41,42^ However, while meaningful radiographic responses and improvements in survival are seen in systemic cancers when targeted agents are selected using the patient’s NGS results, this is distinctly uncommon in patients with high-grade gliomas.^43,44^

The presence of the BBB provides a ready explanation for the failure of many chemotherapy drugs and appropriately selected targeted agents to improve outcomes in patients with CNS malignancies. The BBB initially evolved hundreds of millions of years ago in invertebrates with a distributed nervous system to provide precise control of CNS homeostasis.^3^ This ensured proper neuronal function and also protected the neural tissues from toxins and pathogens. The early BBB was neuronally based, but as the nervous system became more centralized, endothelial cells in blood vessels carrying oxygen and nutrients to the brain assumed responsibility for tightly regulating the movement of ions, molecules, and cells between the blood and the brain. The BBB is comprised of a series of physical, transport, and metabolic properties that are regulated by interactions with vascular, immune, and neural cells. The presence of the BBB in early species and its evolution over time highlight its importance to the CNS. It is exceedingly efficient in isolating the brain from compounds in the bloodstream as evidenced by the fact that it effectively restricts over 98% of drugs currently approved by the Food and Drug Administration (FDA) from entering the brain or spinal cord.^5^

While high-grade gliomas usually have a partially disrupted BBB that allows intravenously administered contrast agents to leak into brain tumors, there are large regions within these tumors that are non-enhancing on MRI but are replete with actively dividing tumor cells. Contrast agents used in neuroimaging, such as gadolinium (molecular weight 204 g/mol), have been specifically designed to identify regions in the brain with minimal changes in BBB integrity. However, most drugs used to treat cancer have physical properties (molecular weight, lipid solubility, charge, pump substrates, etc), that make them much less likely than gadolinium to cross the BBB. Even temozolomide (molecular weight 194 g/mol) has drug concentrations in the brain that are less than 20% of concentrations in the blood.^45^ The efficiency of the BBB also explains why patients whose liver, lung, and other systemic metastases are responding to targeted therapies often have isolated brain and/or leptomeningeal metastases as their most frequent site of relapse.^6,7^ While systemic levels of these drugs result in responses, these agents do not reach the brain in therapeutic concentrations allowing micrometastases to flourish in that environment.

Having potentially effective medications in the pharmacy is of little benefit to patients with primary brain tumors or patients at risk to develop isolated central nervous system metastases if these agents cannot reach the brain in therapeutic concentrations. As a result, it is imperative that future research specifically focuses on novel ways to transiently manipulate the integrity of the BBB. As discussed earlier in this manuscript, many approaches to modify the integrity of the BBB have been and are being explored. Most of these are designed to transiently disrupt the BBB in a focal region of the brain. Unfortunately, most primary brain tumors have infiltrated far beyond what is visible on MRI scans and focal disruptions of the BBB will not address the prevention of brain metastases. An alternative approach might be to use vasoactive peptides administered with active antineoplastic drugs to transiently disrupt the entire BBB in an effort to improve drug delivery to non-enhancing regions of the brain which contain actively growing tumor cells and to potentially prevent the development of CNS metastases in patients with systemic cancers.

Although it still has to be shown that vasoactive peptides can transiently disrupt the BBB in humans, both bradykinin and adenosine appear to effectively accomplish this in rodents. Further studies are required to determine how to translate these findings into humans. Animal data suggests that different administration schedules may be more effective.^16^ In addition, to date no studies have been conducted to determine if combining bradykinin and adenosine might be additive or synergistic in transiently disrupting the BBB. This research would be best conducted in humans given the potential differences between the rodent and human BBB. Furthermore, it would be ideal to quantify the effect of vasoactive peptides on the BBB using noninvasive imaging techniques prior to conducting invasive pharmacokinetic studies of drug concentrations before and after vasoactive peptide administration. Stated another way, if vasoactive peptides are unable to substantially enhance the entry of gadolinium in the brain, it is unlikely that they will increase brain concentrations of chemotherapy or targeted agents sufficiently to result in higher response rates or improved survival.

The study reported in this manuscript employed a trial design to noninvasively screen and develop novel agents and approaches to transiently modify the integrity of the BBB. This trial explored the use of a vasoactive peptide in humans that transiently opens the BBB in rodents. Our underlying assumption was that if this adenosine agonist did not significantly increase in *K*^*trans*^ of gadolinium in a normal brain it would be unlikely to provide a meaningful increase in the CNS penetration of systemically administered drugs. This approach would permit sequential evaluations of doses, schedules, and combination approaches before embarking on the necessary but much more complicated, invasive, and expensive pharmacokinetic studies.

In this study, we set a high bar for success—a 10-fold increase in the *K*^*trans*^ for gadolinium after the administration of regadenoson. We did not meet that bar. It is important to note that our average measured *K*^*trans*^ (0.0013 min^−1^) in white matter was substantially lower than our expected values of around 0.004 min^−1^, which could be explained by several factors. First, the permeability of normal white matter is controversial and highly variable in the literature. While we chose values reflecting a higher *K*^*trans*^^,29,31,33,35^ other studies have estimated *K*^*trans*^ in the intact BBB anywhere below 0.005 min^−1^. ^46–48^Notably, *K*^*trans*^ is highly dependent on several technical and biological factors, including magnetic field strength, DCE-acquisition parameters, selection of arterial (or venous) input function, effective use of motion correction, the type of pharmacokinetic model used (ie, Pathak vs. Tofts vs. Extended Tofts), and total acquisition time.^48–50^ Additionally, data suggests *K*^*trans*^ in normal white matter is highly dependent on plasma volume fraction, *v*_*p*_, with lower plasma volume fraction resulting in lower *K*^*trans*^, which can lead to more challenging to quantify precisely.^48^ It is conceivable that patients in the current study had a low plasma volume fraction due to previous treatments (eg, radiation) or other factors, resulting in a slightly lower *K*^*trans*^ than we originally theorized.

A secondary endpoint in this study was a greater than 100% increase in Z-score normalized T1-weighted MR signal intensity on T1 subtraction maps following regadenoson administration. We reached a 75% increase in the Z-score suggesting that there might be a pharmacodynamic effect but that this was unlikely to be sufficient to improve drug entry enough to alter clinical outcomes. As a result, further efforts are required to evaluate repetitive dosing of regadenoson and to test combinations of adenosine and bradykinin analogs. Although we have many potentially effective chemotherapeutic and targeted agents available to treat patients with CNS malignancies, we currently lack the ability to help these agents reach their targets in therapeutic concentrations, especially in non-enhancing regions of the brain. Future research on how to increase CNS drug delivery is desperately required in order to improve the outcomes of patients with primary brain tumors and to reduce the incidence of brain metastases in patients with systemic cancers who are responding to targeted agents.

## Supplementary Material

vdaf041_suppl_Supplementary_Materials

## Data Availability

The authors are committed to making all deidentified data which the conclusions of the paper rely available to readers. This data is currently included in the manuscript and the supplementary files
