# Supplementary material for: Assessing the dose of regadenoson required to transiently alter blood-brain barrier integrity in patients with infiltrating gliomas
Source: Neurooncol Adv. 2025 Feb 15;7(1):vdaf041. doi: 10.1093/noajnl/vdaf041 (PMC11992616; doi:10.1093/noajnl/vdaf041)
Supplement: vdaf041_suppl_Supplementary_Materials [file vdaf041_suppl_supplementary_materials.docx]

**SUPPLEMENTARY INFORMATION:**

1. **Supplementary Text -- Detailed Eligibility and Exclusion Criteria**
2. **Supplementary Tables**

Supplementary Table 1: List of Prohibited Medications

Supplementary Table 2: MRI acquisition specifics

**SUPPLEMENTARY TEXT**

1. **Detailed Eligibility and Exclusion Criteria**

Patients were carefully selected for this non-therapeutic study to minimize risks from the administration of regadenoson. Eligible patients were young, without known cardiac disease, and not taking potentially neurotoxic medications as part of their routine medical care in case regadenoson opened the BBB and allowed these medications to have access to the central nervous system. Furthermore, every effort was made to ensure that selected patients did not have rapidly progressive tumors as this would have complicated the analysis of the efficacy of regadenoson which relied on changes between a baseline scan and the later scan with regadenoson. These important factors heavily impacted the eligibility and exclusion criteria for this study.

Patients eligible for this study were required to have a prior histologic diagnosis of a glioma of any grade, be 18-45 years of age, have a KPS of >80, be able to provide an informed consent, and to undergo an MRI with contrast. There were no restrictions regarding the number of prior therapies or relapses but they were required to have serial routine follow up MRIs that did not reveal progressive disease for over 2 months before entering this study. In addition, they had to be without progressive symptoms or signs of tumor progression. For patients who were on treatment, the following intervals from previous treatments were required to be eligible: 12 weeks from the completion of radiation, 16 weeks from an anti-VEGF therapy, 6 weeks from a nitrosourea chemotherapy, 3 weeks from a non-nitrosourea chemotherapy, 2 weeks or 5 half-lives from any investigational (not FDA-approved) agents, and 2 weeks from administration of a non-cytotoxic, FDA-approved agent (e.g., erlotinib, hydroxychloroquine, etc.). Patients were also required to have adequate organ and marrow function including a creatinine ≤ 1.5 mg/dL or eGFR ≥30 mL/min/1.73 m^2^. Patients allocated to receive regadenoson at the 1.0 mg dose level were required to weigh more than 50 Kg and patients allocated to receive regadenoson at the 1.4 mg dose level needed to weigh more than 70 Kg.

Patients were excluded from this study if they had not recovered from adverse events due to prior anti-cancer therapy (*i.e.*, have residual toxicities > Grade 2) with the exception of alopecia, if they were receiving any other investigational agents, or had a history of hypersensitivity reactions attributed to compounds of similar chemical or biologic composition to regadenoson. In addition, patients were excluded if they had a history, current symptoms or signs of cardiovascular disease including: any ischemic cardiac event (myocardial infarction, coronary revascularization, stable or unstable angina), ischemic or nonischemic cardiomyopathy and/or congestive heart failure, supraventricular tachycardia, atrial fibrillation, and/or atrial flutter, ventricular tachyarrhythmias, severe sinus bradycardia defined as a resting heart rate <40 bpm, symptomatic bradycardia, sick sinus syndromes greater than first-degree AV block, left bundle branch block, and/or presence of a cardiac pacemaker, stenotic valvular heart disease or uncontrolled hypo- or hypertension defined as a systolic blood pressure <90 mmHg or >180 mmHg, respectively. Patients with uncontrolled asthma or seizures, psychiatric illness/social situations that would limit compliance with study requirements, pregnancy or breast feeding, or another uncontrolled concurrent illness were not eligible for this study. In addition, patients taking potentially neurotoxic medications (Supplementary Table 1) were required to discontinue these for more than one week before the injection of regadenoson to be eligible for this study.

1. **Supplementary Table 1: List of Prohibited Concurrent Medications:**

Patients taking potential neurotoxic medications should be off of them for ≥1 week before the injection of regadenoson. These medications include:
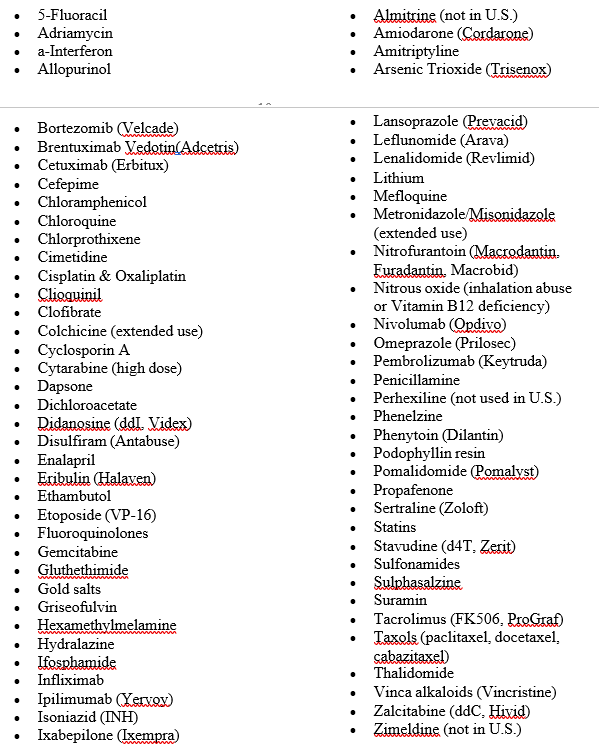


**Supplementary Table 2: MRI Protocol Acquisition Specifics**

|  | **3D T1w**  **Pre^b^** | **Ax 2D FLAIR^j^** | **Ax 2D DWI** | **Ax 2D**  **T2wh,i** | **T1 Map** | **DCE^a^** | **3D T1w Post^b^** |
| --- | --- | --- | --- | --- | --- | --- | --- |
| Sequence | MPRAGE  e,f | TSE^c^ | SS-EPI^g^ | TSE^c^ | 3D-FLASH | 3D-FLASH | MPRAGE^e,f^ |
|  |  |  |  |  |  |  |  |
| Plane | Sagittal or  Axial | Axial | Axial | Axial | Axial | Axial | Sagittal/  Axial |
| Mode | 3D | 2D | 2D | 2D | 3D | 2D | 3D |
| TR [ms] | 2100^m^ | >6000 | >5000 | >2500 | 4-6 | 4-6 | 2100^m^ |
| TE [ms] | Min | 100-140 | Min | 80-120 | Min | Min | Min |
| TI [ms] | 1100^n^ | 2000-  2500^k^ |  |  |  |  | 1100^n^ |
| Flip Angle [Degrees] | 10-15 | 90/≥160 | 90/180 | 90/≥160 | 2/10/15  20/30 | 20 | 10-15 |
| Frequency | ≥172 | ≥256 | ≥128 | ≥256 | 256 | 256 | ≥172 |
| Phase | ≥172 | ≥256 | ≥128 | ≥256 | 128-256 | 128-256 | ≥172 |
| NEX | ≥1 | ≥1 | ≥1 | ≥1 | 2 | NEX=1 (130  Reps)  ~6.5sec/rep | ≥1 |

|  |  |  |  |  |  | (>10 baseline, pre-contrast  points) |  |
| --- | --- | --- | --- | --- | --- | --- | --- |
| Frequency  Direction | A/P | A/P | R/L | A/P | R/L | R/L | A/P |
| FOV | 256 | 240 | 240 | 240 | 256 | 240 | 256 |
| Slice Thickness | ≤1.5mm | ≤4mm^l^ | ≤4mm^l^ | ≤4mm^l^ | 3-5mm | 5mm | ≤1.5mm |
| Gap/Spacing | 0 | 0 | 0 | 0 | 0 | 0 | 0 |
| Diffusion Options^p^ |  |  | *b* = 0,  500, 1000  s/mm^2^  ≥3 directions |  |  |  |  |
| Parallel  Imaging | Up to 2x | Up to  2x | Up to 2x | Up to 2x | Yes-If  Available | Yes-If  Available | Up to 2x |
| Scan Time (Approx) [Benchmarked on 3T Skyra] | 5-10 min  [5:49 for 1mm isotropic] | 4-8 min  [3:22  for 2D FLAIR] | 2-4 min  [1:22 for  3  direction DWI and 3 b-  values] | 4-8 min  [5:10 for dual echo] | < 1 min ea | 8 min | 5-10 min  [5:49 for 1mm isotropic] |

^a^ 0.1 mmol/kg dose injection with a Gadolinium chelated contrast agent. Use of a power injector is desirable at an injection rate of 3-5cc/sec.

^b^ Post-contrast 3D T1-weighted images should be collected with equivalent parameters to pre-contrast 3D T1-weighted images

^c^ TSE = turbo spin echo (Siemens & Philips) is equivalent to FSE (fast spin echo; GE, Hitachi, Toshiba) ^d^ FL2D = two-dimensional fast low angle shot (FLASH; Siemens) is equivalent to the spoil gradient recalled echo (SPGR; GE) or T1- fast field echo (FFE; Philips), fast field echo (FastFE; Toshiba), or the radiofrequency spoiled steady state acquisition rewound gradient echo (RSSG; Hitachi). A fast gradient echo sequence without inversion preparation is desired.

^e^ MPRAGE = magnetization prepared rapid gradient-echo (Siemens & Hitachi) is equivalent to the inversion recovery SPGR (IR-SPGR or Fast SPGR with inversion activated or BRAVO; GE), 3D turbo field echo (TFE; Philips), or 3D fast field echo (3D Fast FE; Toshiba).

^f^ A 3D acquisition without inversion preparation will result in different contrast compared with MPRAGE or another IR-prepped 3D T1-weighted sequences and therefore should be avoided.

^g^ In the event of significant patient motion, a radial acquisition scheme may be used (e.g. BLADE [Siemens], PROPELLER [GE], MultiVane [Philips], RADAR [Hitachi], or JET [Toshiba]); however, this acquisition scheme is can cause significant differences in ADC quantification and therefore should be used only if EPI is not an option. Further, this type of acquisition takes considerably more time.

^h^ Dual echo PD/T2 TSE is optional for possible quantification of tissue T2.

^i^ Advanced sequences can be substituted into this time slot, so long as 3D post-contrast T1-weighted images are collected between 4 and 8 min after contrast injection.

^j^ 3D FLAIR is an optional alternative to 2D FLAIR, with sequence parameters as follows per EORTC guidelines: 3D TSE/FSE acquisition; TE=90-140ms; TR=6000-10000ms; TI=2000-2500ms (chosen based on vendor recommendations for optimized protocol and field strength); GRAPPA≤2; Fat Saturation; Slice thickness ≤ 1.5mm; Orientation Sagittal or Axial; FOV ≤ 250 mm x 250 mm; Matrix ≥ 244x244.

k Choice of TI should be chosen based on the magnetic field strength of the system (e.g. TI ≈ 2000ms for
